# Moo19 and B2: Structures of *Schitoviridae* podophages with *T* = 9 geometry and tailspikes with esterase activity

**DOI:** 10.1126/sciadv.adt0022

**Published:** 2024-12-18

**Authors:** Sundharraman Subramanian, Silje M. Bergland Drarvik, Kendal R. Tinney, Sarah M. Doore, Kristin N. Parent

**Affiliations:** ^1^ Department of Biochemistry and Molecular Biology, Michigan State University, East Lansing, MI 48824, USA.; ^2^ Department of Integrative Biology, Michigan State University, East Lansing, MI 48824, USA.; ^3^ Department of Microbiology and Cell Science, University of Florida, Gainesville, FL 32611, USA.

## Abstract

Podophages are, by far, the least well studied of all the bacteriophages. Despite being classified together due to their short, noncontractile tails, there is a huge amount of diversity among members of this group. Of the podophages, the N4-like *Schitoviridae* family is the least well studied structurally and is quite divergent from well-characterized podophages such as T7 and P22. In this work, we isolate and fully characterize two members of the *Schitoviridae* family by cryo–electron microscopy, genetics, and biochemistry. We describe the capsid features of Moo19 and B2, including a decoration protein. In addition, we have fully modeled the tail machinery for both phages and identify proteins with esterase activity. Genetic knockouts of the host reveal factors specific for host attachment including key modifications to the O-antigen on the lipopolysaccharide. Moo19 and B2 are both *Schitoviridae* members, yet some distinct differences in the genome and structure place them into distinct clades.

## Teaser

Two structures of *Shigella* podophages with *T* = 9 geometry solved by cryo-EM.

## INTRODUCTION

Viruses that infect bacteria, bacteriophages, or phages, are highly diverse and are found in a variety of environments. Estimates suggest that over 10^31^ viruses and bacteriophages exist in the biosphere (*1*, *2*), and an increase in phage isolation methods or “hunting” activities has greatly expanded our understanding of their morphological and biochemical diversity (*3*–*6*). The vast majority of known phages are double-stranded DNA (dsDNA)–containing tailed phages, and their morphology has been historically described by the tail machinery: Phages with long, contractile tails (myovirus) and long, noncontractile tails (siphovirus) are generally the more commonly found varieties in nonmarine environments (*5*, *7*). By contrast, phages with short, noncontractile tails (podovirus) tend to be less abundant. In general, we know very little about how podophages infect their hosts (*8*), although some examples, such as for the *Salmonella* phage P22 (*9*–*11*), the *Escherichia coli* phage T7 (*12*–*14*), and the *Shigella* phage Sf6 (*15*–*17*), have been well characterized genetically, structurally, and biochemically.

The term podophage encompasses a very wide distribution of phage types. There are some major differences among podophages such as vastly different overall morphologies, minimal sequence homology of proteins, and deviations in overall life cycle (*8*). The well-studied podophages T7, P22, Sf6, and CUS-3 share some structural features—their capsids all have isometric *T* = 7 capsid geometry (meaning 415 copies of the major capsid protein) that encapsidate genomes with lengths roughly around 40 kb. However, the tail of T7 is distinct, with tail fibers and an “inner core” of capsid proteins that form a tube for dsDNA delivery into the host. T7 also requires a phage-encoded polymerase for infection (*12*). By contrast, Sf6, P22, and CUS-3 have extended complexes of the C-terminal portions of 12 portal proteins instead of a T7-like inner core that facilitates DNA delivery (*18*). In addition, Sf6, P22, and CUS-3 have enzymatic tailspike proteins, tail needles, and three types of ejection proteins that are released into the host during infection, with no virion-associated polymerase (*19*). Other podophages are wildly different in terms of both capsids and tails. *Bacillus* phage φ29 has a prolate capsid, a much smaller genome of ~20 kilo–base pairs (kbp), a tail assembled from appendage proteins, and a tail tube (*20*). *E. coli* N4 has a much larger genome and a capsid with a relatively rare *T* = 9 geometry composed of 535 capsid proteins and an unusually large virion-associated RNA polymerase. These differences place N4 in a group most distantly related to the other podoviruses, leading to a recently proposed reclassification to a family called *Schitoviridae* (*21*). The N4-like phages are not well understood.

In the past several years, our laboratory has begun characterizing the diversity and abundance of *Shigella* phages isolated from various environments. This work began with the observation that, despite over 100 years of research on bacteriophage, very few *Shigella* phages have been isolated and characterized in general, relative to hundreds (if not thousands) of reports of other enteric phages (*22*). Phage hunting efforts have led to a plethora of recently found and characterized *Shigella* phages (*23*–*28*) and have greatly expanded knowledge of these phages and their life styles. Very few *Shigella* podophages were isolated as part of this effort. Since we began our hunting program in 2016, only a single *Shigella* podophage was identified from thousands of samples (*25*). The *Shigella* podophage HRP29 has a *T* = 7 capsid, a small genome (~40 kb), and a tail that is a hybrid between T7-like and Sf6-like phages (*29*).

In this study, we tried to expand our understanding of podophages by using a different “bait” *Shigella*. We isolated and characterized two *Shigella* podophages that have notably different features when compared with classical podophages and instead are most closely related to phage N4. We describe the isolation and characterization of phages Moo19 and B2, including whole-genome sequencing and analysis, cryo–electron microscopy (cryo-EM) structure determination, mass spectrometry, host range studies, and enzymatic assays of the tail proteins. Both phages have large genomes (~72 kbp) and corresponding capsid size, likely forming a *T* = 9 capsid geometry, which appears common among a subset of Moogle-like *Shigella* myophages (*24*, *25*) but, to date, has not been observed in *Shigella* podophages. Our work shows that these phages have divergent decoration proteins and unique tail structures, and encode and package their own phage-dependent RNA polymerase, and the tails, which display esterase activity, are dependent on specific O-antigen modifications of lipopolysaccharide (LPS) for entry.

## RESULTS AND DISCUSSION

Phage hunting activities as part of the graduate curriculum at Michigan State University (East Lansing, MI) and as part of a high school outreach activity at Lincoln Southwest High School (Lincoln, NE) have been ongoing in our laboratory since 2016 (*23*). Initially, we found only one podophage, from over 2000 samples collected and processed, making *Shigella* podophages very uncommon. HRP29, the single podophage example, to date, was recently described and was found to have a ~40-kb genome and typical *T* = 7 capsid geometry yet a hybrid tail that has components similar to both phages P22 and T7 (*29*). In this work, we used a different bait bacterial strain and isolated two *Shigella* podophages from Lincoln, Nebraska that differ greatly from HRP29: Moo19 and B2.

**Fig. 1.**
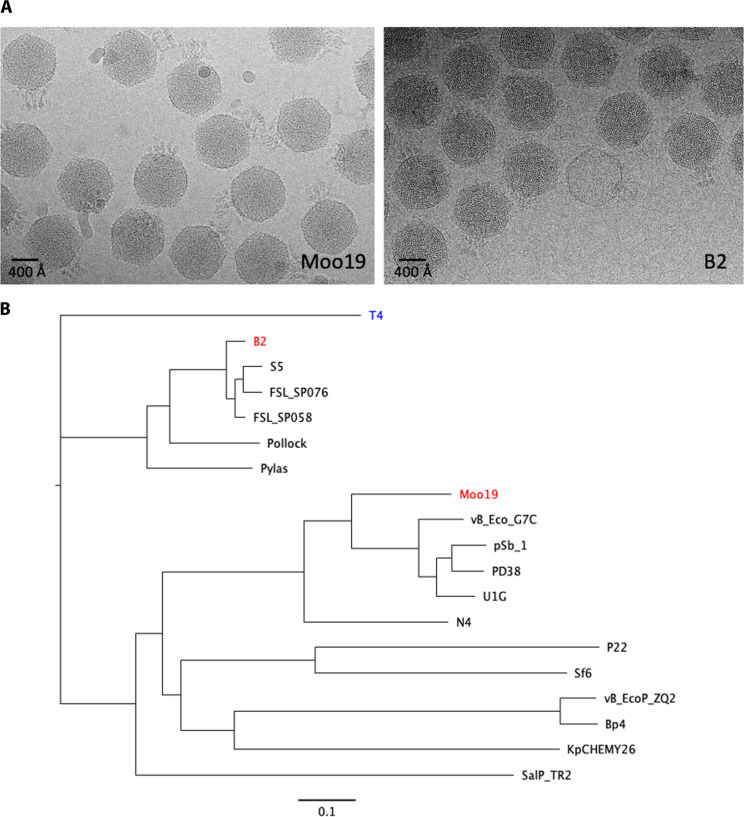
Initial characterization of Moo19 and B2. (**A**) Representative cryo-micrographs of Moo19 and B2. (**B**) Phylogenetic trees. Whole-genome sequence phylogenetic tree of related podophages, except for myovirus phage *E. coli T4* (blue) used as an outgroup. *S. flexneri* phages of interest Moo19 and B2 are shown in red. Other phages compared in the tree infect bacterial hosts from the family *Enterobacteriaceae* including *S. flexneri* (pSb-1, Sf6), *E. coli* (N4, vB_EcoP_G7C, vB_EcoP_ZQ2, U1G, PD38, and Pollock), *Salmonella enterica* (P22, S5, FSL-SP058, FSL-SP076, and SalP_TR2), *K. pneumoniae* (Pylas and KpCHEMY26), and *Enterobacter cloacae* (Bp4). The scale bar demonstrates a 0.1 nucleic acid substitution per nucleotide site.

Moo19 was isolated from a water sample collected from a cow pasture in 2019, and B2 was isolated from a freshwater pond 2 years later in 2021. After initial plaque isolation and purification, phages were amplified and visualized by negative stain EM and cryo-EM. Initial inspection of the cryo-micrographs (Fig. 1A) showed that the overall morphology of Moo19 and B2 differed from both the recently reported podophage HR29 and the well-studied *Shigella* podophage Sf6. Particularly interesting was the highly decorated tail structure and the substantially larger capsid. Given the distinct differences in these phages from previously isolated *Shigella* podophages, we pursued in-depth characterization.

### Phylogenetic analysis

We performed whole-genome sequencing and compared the Moo19 and B2 genomes to other published phages (Fig. 1B). The Moo19 genome is 72,458 kbp, and B2 is ~71,028 kbp. Whereas the best characterized *Shigella* podophage is the P22-like phage Sf6 (~40 kbp), neither Moo19 nor B2 showed significant genomic similarity to the *Lederbergvirus* genus. Genome size is often closely linked to capsid geometry as a larger genome needs a larger capsid volume to be contained (*6*). Analysis showed that Moo19 was more closely related to *E. coli* phage N4, one of the few described *T* = 9 podophages and a representative member of family *Schitoviridae*.

The subfamily *Enquatrovirinae* within the family *Schitoviridae* is currently divided into three genera: the Enquatroviruses, typified by N4; the *Gamaleyaviruses*, typified by G7C; and the small genus Kaypoctaviruses, typified by KP8. All are predominantly *E. coli* phages, with the exception of *Klebsiella pneumoniae*–infecting Kaypoctaviruses and the Gamaleyavirus pSb-1, which infects *Shigella boydii*. Whereas the other lone *Shigella* phage pSb-1 is a close relative of G7C, *Shigella* phage Moo19 is distinct from this and all other Enquatrovirinae clades, suggesting that it is a distinct species.

Although B2 is also a member of the *Schitoviridae* family, it belongs to the *Humphriesvirinae* subfamily, which is also divided into three genera: the Ithacaviruses, which primarily infect *Salmonella* (*30*); the Pollockviruses, of which *E. coli* phage Pollock is the single member (*31*); and the Pylasviruses, which infect *Klebsiella* (*32*). Of these genera, *Shigella* phage B2 clusters with other Ithacaviruses. To our knowledge, *Humphriesvirinae* have not been structurally characterized beyond negative stain TEM (*33*).

Because N4 is a podophage that encapsidates its own RNA polymerase (*34*), and the closest relative to Moo19, we tested whether Moo19 and B2 encapsidate their own RNA polymerases as well. We used mass spectrometry to identify proteins associated with high-titer, CsCl-purified phages for both Moo19 and B2 to identify if the putative RNA polymerase gene products were part of the mature virions (Table 1). The RNA polymerases gp67 (Moo19) and gp30 (B2) were positively identified from trypsin-digested fragments with over 95% confidence, indicating that RNA polymerase is associated with mature virions. Blast alignment showed that Moo19’s gp67 shares 67.1% identity with N4’s RNA polymerase. By contrast, B2’s gp30 is much more divergent, sharing only 25.3% identity to N4’s RNA polymerase.

**Table 1.** Mass spectrometry results of B2 and Moo19. Bold font means that the protein has an atomic model fitted into the cryo-EM map. “N/A” means that no homolog was found in the genome or in the mass spectrometry data.

Protein role	Moo19, MW	B2, MW
V RNA polymerase	gp67, 378 kDa	gp30, 411 kDa
**Tailspike protein**	gp82, 118 kDa	gp49, 44 kDa (part of complex with *gp48*)
**Tail fiber**	N/A	gp48, 83 kDa (part of complex with *gp49*)
Hypothetical protein, similar to N4’s gp53	gp70, 96 kDa	N/A
**G7C gp66-like tail protein**	gp81, 93 kDa	N/A
**Portal protein**	gp76, 85 kDa	gp39, 79 kDa
**Adaptor**	gp68, 70 kDa	gp50, 27 kDa
**Major capsid protein**	gp73, 44 kDa	gp36, 42 kDa
**Tail tube**	gp71, 30 kDa	gp34, 23 kDa
**Decoration protein**	gp28, 28 kDa	gp45, 9 kDa
Predicted structural protein	gp83, 27 kDa	N/A
Predicted structural protein	gp69, 16 kDa	N/A
Hypothetical protein, similar to N4’s gp31	gp46, 8 kDa	N/A

### Structure analysis

#### Virion and capsid

**Fig. 2.**
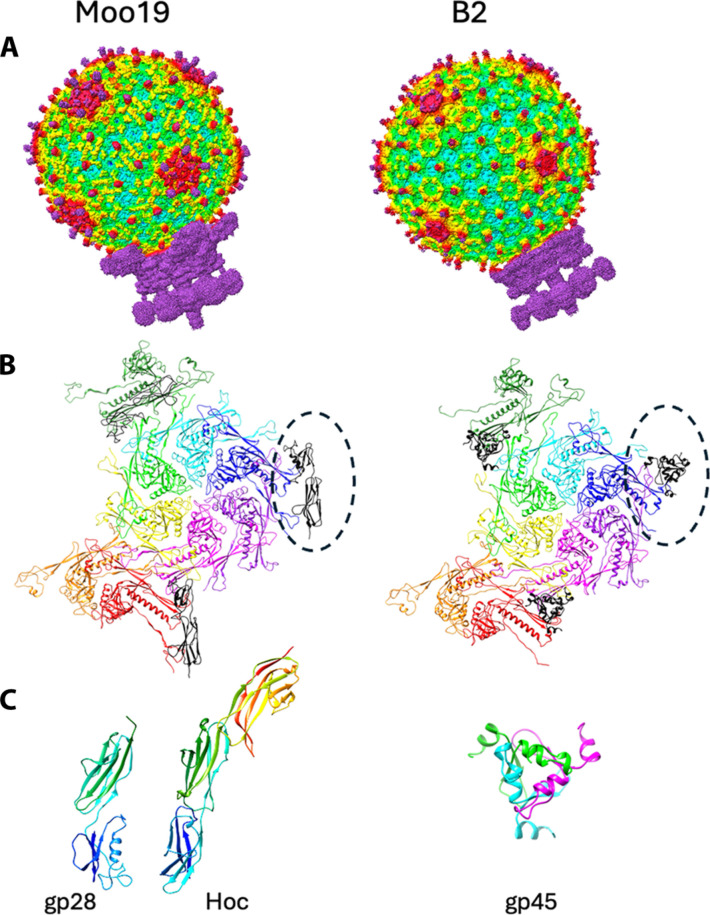
Structure of Moo19 and B2 virions, capsid proteins, and decoration proteins. (**A**) Surface renderings of asymmetric reconstructions for both Moo19 and B2 whole virions. (**B**) Ribbon models of the capsid asymmetric unit including nine chains of the major capsid protein (rainbow colored by subunit) and the decoration proteins depicted as black ribbons and highlighted by a dotted oval. (**C**) Monomer of Moo19’s decoration protein gp28 compared with phage T4’s Hoc [PDB ID: 3SHS (*39*)]. Each decoration protein monomer is color coded in a rainbow by residue, where the N terminus is blue and C terminus is red. B2’s decoration protein (gp45) is a homotrimer with each chain individually colored.

To date, no high-resolution structure has been published for the entire N4-like virions. To characterize Moo19 and B2, we used cryo-EM of mature virions and performed three-dimensional (3D) image reconstructions of the entire virions (Fig. 2A). We also analyzed the data by imposing icosahedral symmetry to visualize the capsids at high resolution (3.6 and 3.4 Å for Moo19 and B2, respectively). Both capsids have a *T* = 9 geometry (see Fig. 2B for a depiction of the capsid proteins that comprise the asymmetric unit). The major capsid protein of each has the HK97-like fold that is present in most, if not all, dsDNA tailed phages (*35*). Using DALI (*36*), both the major capsid proteins of Moo19 and B2 are most similar to Ralstonia phage GP4 (*37*), another *T* = 9 podophage with an RMSD (root mean square deviation) of 3.0 and 3.2 Å, respectively.

Both Moo19 and B2 capsids have additional surface proteins similar to what is referred to as phage “cement” or “decoration” proteins that often provide capsid stability (*38*). The decoration proteins of both Moo19 and B2 bind the capsid proteins at the same quasi-sixfold symmetry axes in both phages (Fig. 2B) but have notably different folds (Fig. 2C). Moo19’s decoration protein (gp28) displays an immunoglobulin (Ig) domain–like structure that is similar to Hoc in phage T4 (*39*). The crystal structure of Hoc [Protein Data Bank (PDB) ID: 3SHS] shows that each monomer (304 amino acids) folds as three Ig domains that come together as a trimer to form a spike on the surface of phage T4’s capsid. The cryo-EM density map of Moo19 was sufficient to clearly model the first 183 amino acids of 273 total residues in gp28. The C-terminal portion is likely also an Ig-like domain that forms the trimer spike similar to Hoc, but the resolution of this region was too poor to accurately model. By contrast, B2’s decoration protein (gp45) is only 99 amino acids. It also forms a homotrimer but is quite unlike Hoc or gp28. Instead, gp45 shares a high sequence identity (88 to 96%) with *Klebsiella* phage MY01 (GenBank accession no. WQY99646.1) and *Salmonella* phage SP154 (GenBank accession no. WIC41542.1) head fiber proteins. To our knowledge, there are no reported phage structures that have structural homology to B2’s gp45. There are some examples of phage decoration proteins that have “knotted α-helical” folds [see (*38*) and (*40*) as a review]. However, B2’s gp45 had poor alignment with these helical decoration proteins and did not share a recognizable topology. Ralstonia phage GP4’s decoration protein binds in a very different capsid location and has a completely different fold to both Moo19 and B2 (*37*). Whereas the capsids of Moo19, B2, and GP4 are highly similar, it seems that the capsid decoration proteins may stabilize weak points in the capsids in different ways. Alternatively, these proteins may not have capsid stabilization properties at all and instead serve another, as of yet, unknown function.

#### Tail assembly

**Fig. 3.**
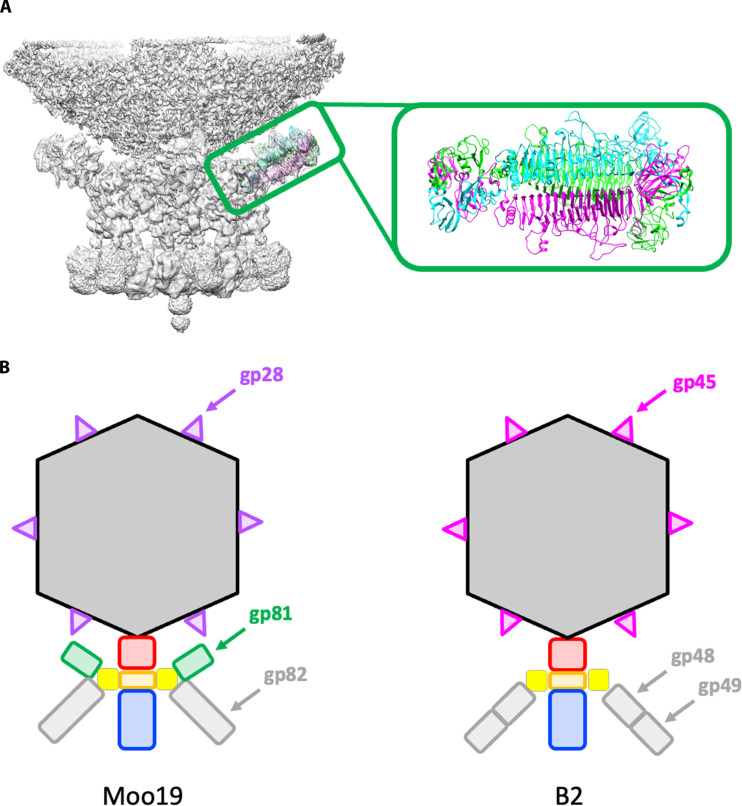
Differences in Moo19 and B2 virions. (**A**) C1-symmetrized focused reconstruction of the Moo19 tail appendage with a trimer of the G7C gp66-like tail (gp81) protein docked. Gp81 is a homotrimer, and each strand is colored individually in cyan, green, and magenta. (**B**) Cartoon representations of Moo19 and B2 highlighting the three key differences in the virions. This includes (i) the addition of gp81 shown in green for Moo19, (ii) a single gene (gp82) for the tailspikes shown in gray for Moo19 and a complex of two gene products in B2 (gp48/gp49), and (iii) variations in the fold of the decoration proteins; gp28 shown in purple for Moo19 and gp45 shown in magenta for B2.

To initially characterize the tails of Moo19 and B2, we analyzed the asymmetric reconstructions of the entire virion and a focused reconstruction of the tail alone using C1 symmetry. Some critical features differed between the two structures. Moo19 has an extra side appendage (composed of gp81, which is observed in the mass spectrometry data shown in Table 1). Gp81 is similar to tail protein gp66 in *E. coli* phage G7C (*41*). Unfortunately, the resolution we obtained using a completely asymmetric reconstruction of the tail alone of the virion was too low (4.3 Å) to fit this structure de novo. Furthermore, using focused reconstruction methods did not improve this resolution enough to reliably model this protein, likely due to flexibility in how gp81 interacts with its binding partner. Therefore, we used AlphaFold2 (*42*) to predict the fold of gp81, and the predicted model was docked into the map (Fig. 3A). There is no homolog predicted in the genome, no similar protein seen in the mass spectrometry data, and no corresponding density observed in the B2 reconstruction. In G7C, this appendage is hypothesized to expand the host range; however, Moo19 and B2 have the same host range, so the function of gp81 in Moo19 is unclear at this time. Three major structural differences (see cartoon representation in Fig. 3B) include (i) the lack of the tail appendage, (ii) a single tailspike for Moo19 and a complex of two proteins for B2 (see next two paragraphs), and (iii) the completely different decoration proteins. These key differences lend support to the phylogenetic analysis that shows Moo19 and B2 are in different clades (Fig. 1).

**Fig. 4.**
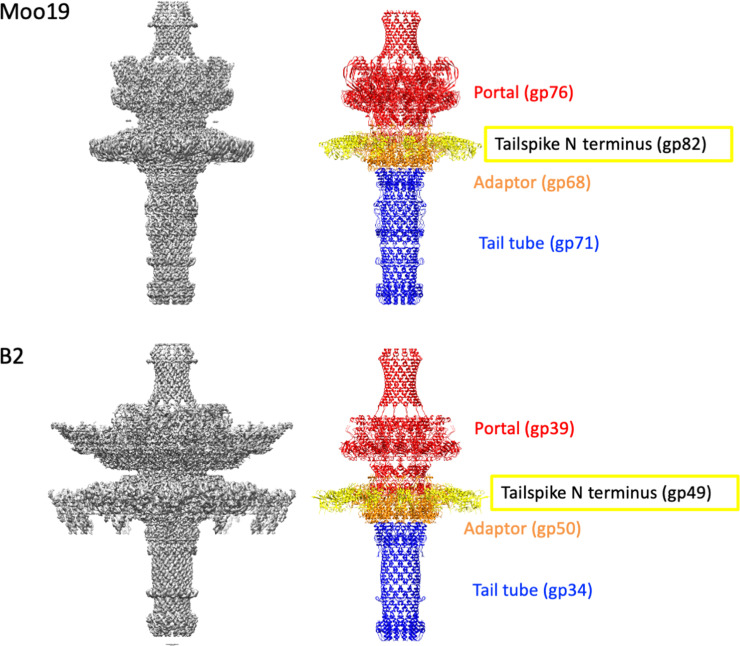
Moo19 and B2 tail machines. (Left) Cryo-EM density maps of the C12-symmetrized tail assemblies for Moo19 and B2. (Right) Atomic models built from the EM density map. The portal shown in red was modeled from residues 17 to 675 of 760 total for Moo19 and residues 182 to 679 of 706 total for B2. The N-terminal portions of tailspike shown in yellow (residues 10 to 74 per monomer in each trimer) were also modeled. The adaptor proteins were largely complete with all 234 residues modeled for Moo19 and residues 2 to 233 of 234 total for B2. Last, the tail tube shown in blue was modeled for residues 4 to 278 of 279 for Moo19 and residues 3 to 200 of 206 for B2.

To further resolve features of the tail machinery, we used focused reconstructions of the tails as previously described (*29*). We were able to clearly model several proteins with our C12-symmetrized maps including most residues for the portal proteins, tail adaptor proteins, and tail tubes for both Moo19 and B2 (Fig. 4). In addition, we were able to model the N-terminal residues of the tailspike proteins for both Moo19 and B2 but not the full-length proteins using the cryo-EM data as these are likely highly flexible at the linkage site. Last, we did observe very weak density at the distal tail tube, likely where a “plug” protein would be found to keep the dsDNA genome from leaking out of the virions. However, given the low resolution, we could not reliably model this protein nor determine its identity. The overall tail machine looks somewhat similar to that of *Pseudomonas* phage DEV [see the work of Cingolani and colleagues (*43*)]. Notable differences are the lack of density around Moo19 and B2 portal protein, whereas DEV has a ring of gp72 protein encircling its portal. In addition, Moo19 and B2 have C12 symmetry and not the proposed C15 symmetry of DEV. The tail tube of Moo19 (gp71) and the tail tube of B2 (gp34) both share a high sequence homology (>80%) with a number of phages that infect enteric hosts such as *Shigella*, *Salmonella*, *Escherichia*, *Klebsiella*, etc. However, most, if not all, of these examples are from metagenomic studies and the genes are annotated as “hypothetical” or “predicted structural protein.” It is likely that this tail tube assembly is highly conserved among *Schitoviridae* members, but more structural data of divergent phages will be needed to confirm this.

**Fig. 5.**
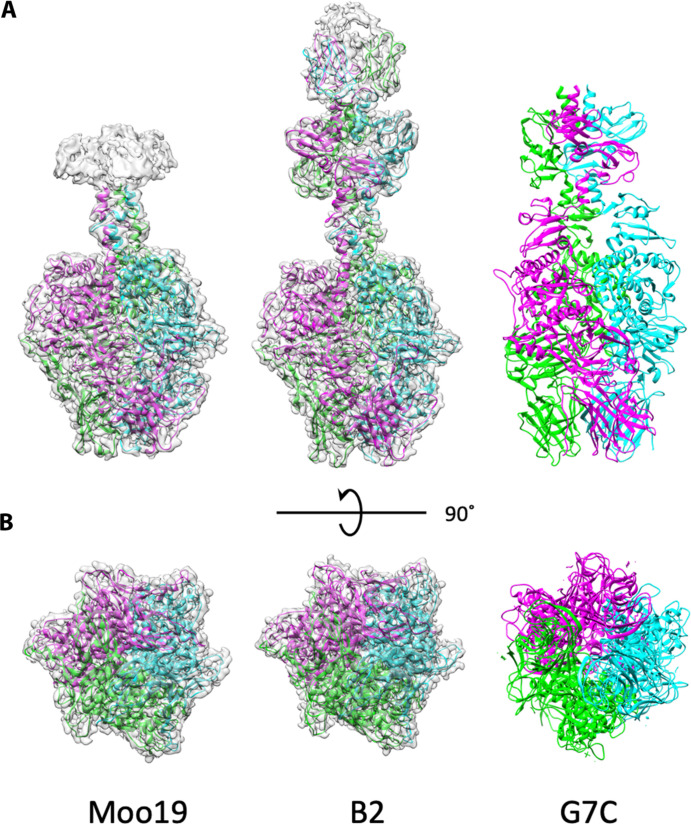
Structure of isolated tailspikes. Cryo-EM density maps of the isolated tailspike proteins (gp82 for Moo19 and a complex of gp48/gp49 for B2) are shown with atomic models fitted in. Each structure is a homotrimer, with individual ribbons colored cyan, green, and magenta. (**A**) Side views. Moo19 and B2 are compared with the tailspike protein in phage G7C [PDB ID: 4QNL (*41*)]. All three display highly similar topology. (**B**) 90° rotation showing the distal end of the trimer that interacts with the host cell surface.

Because we could not resolve the highly flexible side tailspikes in either Moo19 or B2 virions, we purified these components to examine them separately. Genome analysis and mass spectrometry data indicated that the tailspike was a single protein in Moo19 (gp82) and a complex of two proteins in B2 (gp48/gp49) (Table 1). We used a similar approach as described by Subramanian *et al.* (*29*) and purified gp82 alone. We also copurified a complex of gp48/gp49 as previously described (*29*). Using cryo-EM, we solved the structure of the isolated tailspikes to 2.4- and 2.3-Å resolution, respectively (Fig. 5). The tailspikes most closely resemble that of the tailspike in phage G7C (*41*) and differ wildly from the tailspikes in DEV (*43*), which more closely resemble a myophage fiber.

### Host range and receptor analysis

In previous phage isolation efforts, we often observe that *Shigella* phage isolates can have broad host range capability (*24*). *Shigella* phage Sf22 is especially broad as it can infect every species, even across multiple serotypes. Some phages such as Sf21 could even infect across genera, efficiently infecting both *Shigella* and *E. coli*. However, some *Shigella* phages have an extremely narrow host range. For example, HRP29 can only infect *Shigella flexneri* with a highly specific serotype (*25*). Both Moo19 and B2 were isolated on *S. flexneri* strain CFS100 (serotype 2a_2_). To test whether their host range was broad or narrow, we plated these phages against a variety of *Shigella*, *E. coli*, and *Salmonella* strains available in the laboratory (table S1). Moo19 and B2 were only able to infect CFS100, indicating a very narrow host range.

Moo19 and B2 are similar to bacteriophage N4—these are all *T* = 9 podophages, which encapsidate their own polymerases—a hallmark of this phage family. However, Moo19 and B2 likely differ in terms of host attachment and entry mechanisms. It has been well established that phage N4 uses an inner membrane protein NrfB for infection (*44*, *45*) and an outer membrane protein (Omp), NrfA, which interacts with the phage tail sheath (*46*). In addition, the enterobacterial common antigen (ECA) has been implicated as important for phage N4 entry (*47*). Recently, a work has shed light that the NfrA-NfrB system produces a unique exopolysaccharide, also important for N4 adsorption (*48*). *Shigella* species CFS100 lacks an intact NfrA-NfrB system (*49*). Therefore, the attachment and entry mechanisms of Moo19 and B2 likely differ from those of N4 as CFS100 is the host.

Many *Shigella* phages use LPS as the initial and reversible primary receptor (*50*, *51*) and a variety of Omps as the secondary, irreversible receptors (*15*, *52*). In addition, ECA has been shown as also important for N4 and non–N4-like podophages, such as *Salmonella* phage P22 (*11*). For this work, we systematically tested the relevance of each of these components for Moo19 and B2 entry into *Shigella*. We screened Moo19 and B2 against a library of LPS, ECA, and Omp knockouts to determine if these phages require any of these components for successful attachment and entry and whether the entry scheme is different from that of N4 (see table S2 for a list of the strains used). We used quantitative plaque assays (as described in Materials and Methods) to determine if any of these genes affected the life cycle of Moo19 and B2. Productive infections produce plaques, and nonproductive infections do not.

**Fig. 6.**
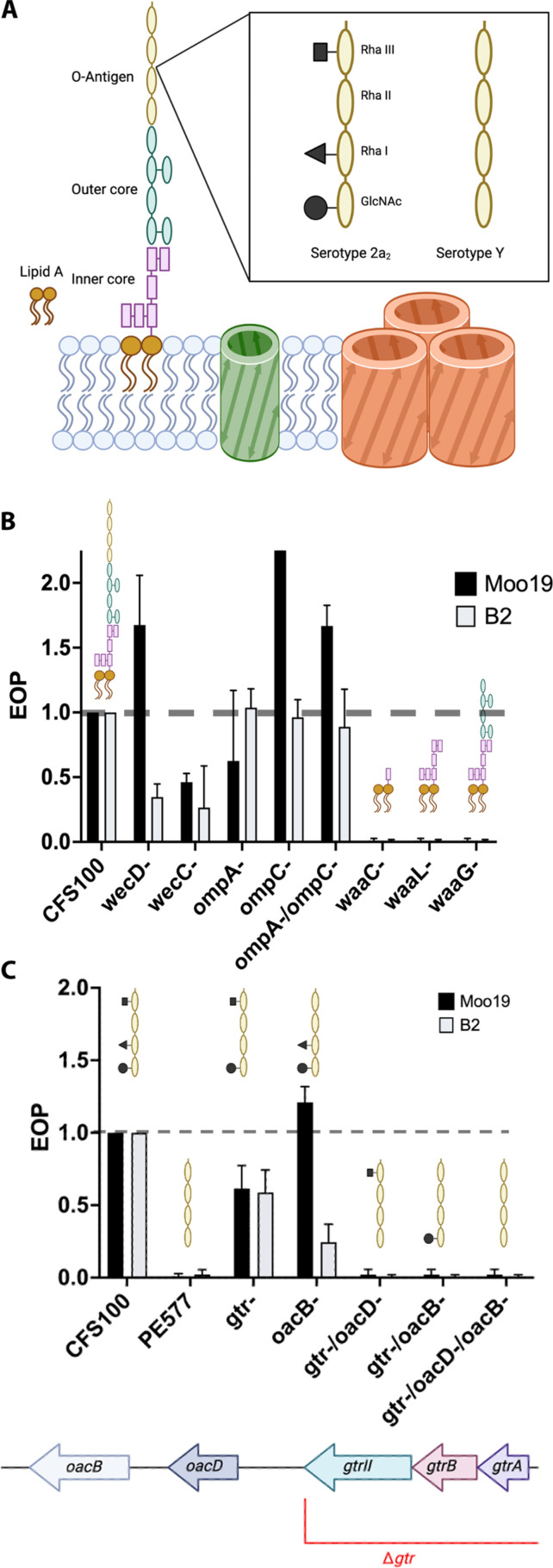
EOP data for a variety of cell surface modifications resulting from genetic knockouts. (**A**) Cartoon schematic of the surface of *S. flexneri* strain CFS100. (**B**) EOP data for ECA knockouts and truncated forms of LPS. (**C**) EOP data with O-antigen modifications. For all panels, EOP was determined by dividing the resulting phage titer on strains with genetic deletions by the titer on the permissive host (CFS100). An EOP of 1.0 means that there is no change in the phages’ ability to infect a strain. Error bars reflect the SD from at least three biological replicates.

We began with genes associated with known attachment mechanisms for *Shigella* phages (Fig. 6A). We narrowed down if Omps, ECA, or LPS were essential (Fig. 6B). Deletions of *wecD*, which is required for ECA synthesis, had no effect on Moo19 infection and only a mild effect on B2 production. In addition, deletion of *wecC*, another gene required for ECA synthesis, had only a mild effect on production of both phages. Together, these results indicate that the ECA molecule is not essential for infection by these phages. Next, we tested Moo19 and B2 for their dependence on OmpA and or OmpC. A previous work has shown that both OmpA and OmpC can act as receptors for *Shigella* phage Sf6 (*15*). In addition, *Shigella* phages Sf22 and KRT47 have a strong reliance on OmpC (*26*). Neither individual nor combination knockouts of *ompA* and/or *ompC* had any effect on plaque formation, indicating that these Omps are not essential for Moo19 or B2 infection.

Next, we tested deletions of *waaC*, *waaL*, or *waaG*, which are truncations to create progressively shorter versions of LPS (Fig. 6B). CFS100Δ*waaC* is a strain with a “deep rough” version of LPS that lacks most of the inner core, the entire outer core, and the O-antigen. The CFS100Δ*waaL* deletion has a “rough” version of LPS that lacks the outer core and the O-antigen but has an intact inner core. Last, CFS100Δ*waaG* is a “semirough” version of LPS that has intact inner and outer cores but lacks the O-antigen. All three knockouts completely inhibited production of Moo19 and B2, indicating that the entire LPS molecule is critical for infection. Therefore, the O-antigen appears to be the essential part of the molecule as knockouts that retain the other major parts of LPS are not viable for Moo19 and B2 infection without the O-antigen.

We then tested the specificity of Moo19 and B2 for O-antigen decoration (Fig. 6C). The host, CFS100, which was originally used as bait for the phage hunting activity, has serotype 2a_2_ LPS, which means there are three modifications present—an O-acetyl group on *N*-acetylglucosamine (GlcNAc), a glucosyl group on Rha I, and a O-acetyl group on Rha III (*53*). By contrast, our typical strain used for phage hunting, PE577, is serotype Y and has a minimal O-antigen unit, with no modifications (*49*, *53*). Neither Moo19 nor B2 was able to infect PE577, indicating that some, or all, of the three serotype 2a_2_ modifications to the O-antigen are needed for host recognition and attachment.

We systematically made knockouts, both individually and in combination, to modify the CFS100 LPS and determine which of the three decorations are important for Moo19 and B2 entry (Fig. 6C). First, we created a knockout to assay the effect of the glucosyl group on Rha I. The *gtr* locus encodes three genes, *gtrII*, *gtA*, and *gtrB*, needed to make this modification (we named this knockout “*gtr*-”). Removing this entire locus resulted in a partial rather mild effect on infection. The *oacB* gene is needed to add the O-acetyl group on Rha III. This deletion had no effect on Moo19 infection yet a partial effect on B2 entry, indicating some critical differences in tail structure between the phage that governs cell recognition. Next, we generated a CFS100*gtr*-Δ*oacD* mutant, which lacks the enzyme needed to add the O-acetyl group on GlcNAc in addition to the missing *gtr* locus. This mutant was completely defective for phage entry for both Moo19 and B2. Unfortunately, despite our best efforts, we were unsuccessful in generating a CFS100Δ*oacD* individual knockout, so we could not assay the effects the *oaB* gene solo.

### Moo19 and B2 tailspike proteins display esterase activity

**Fig. 7.**
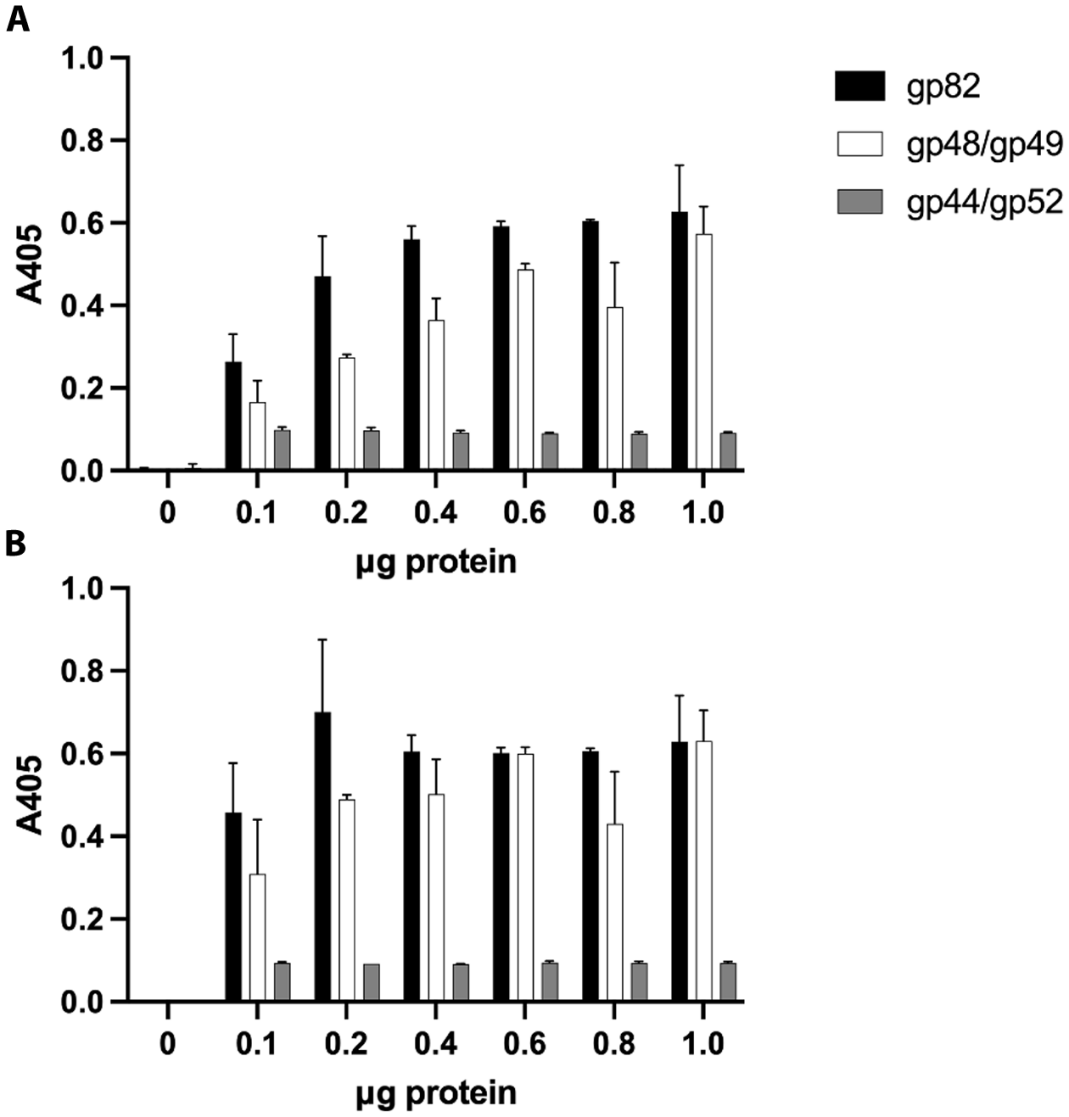
Moo19 and B2 tailspikes display esterase activity. (**A**) Absorbance measured immediately after mixing. (**B**) Absorbance measurement 5 min after mixing. Error bars display the SD from three data points.

Given the genetic data described above, we asked what moiety on the LPS do the tailspikes interact directly with. A previous work on related phage G7C showed that the tailspike proteins act on the LPS molecule as an esterase (*41*). Therefore, we tested the purified Moo19 tailspike gp82 and the complex tailspike from B2, gp49/49, for esterase activity using a colorimetric assay. Briefly, we exposed increasing amounts of each tailspike to 4-nitrophenyl acetate and monitored color change via absorbance at a wavelength of 405 nm. Positive esterase activity would result in a visibly yellow color and increased absorbance with time. Both tailspikes exhibited a rapid, dose-dependent color change, indicating esterase activity (Fig. 7). As a negative control, we also assayed a tail protein complex from podophage HRP29 (gp44/gp52) that does not have esterase activity (*29*), and as expected, no change in absorbance was observed.

### LPS interaction is reversible

**Fig. 8.**
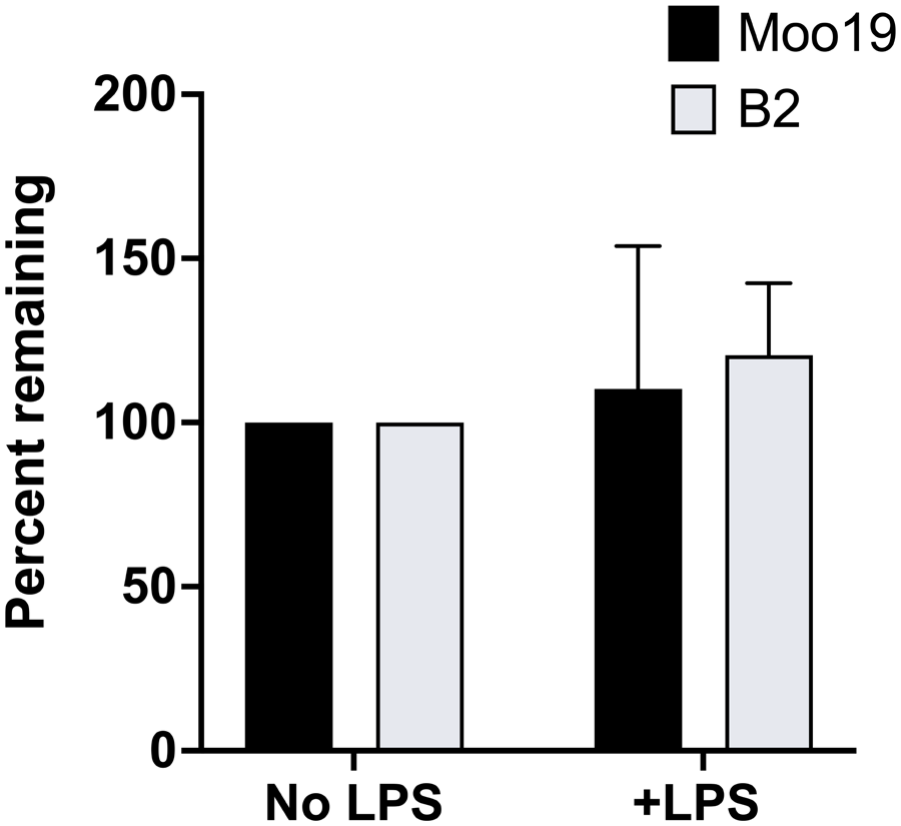
In vitro genome ejections. Percent remaining particles was determined by dividing the resulting phage titer after treatment with LPS by the resulting phage titer after treatment with buffer only. Error bars reflect the SD from at least three biological replicates.

We incubated phage with purified LPS as described previously (*15*, *52*, *54*), assayed virions remaining after incubation, and compared plaque-forming units with those incubated in a no-LPS control to determine if free LPS inactivates Moo19 or B2. No significant change in plaque-forming units is an indication that the phages are interacting reversibly with LPS and still retain their genomes. Alternatively, if LPS alone was enough to trigger genome release or inactivate particles as it is for some other phages such as P22 (*55*) and 9NA (*56*) and related G7C (*41*), we would expect to see a drastic reduction in infectious particles as the empty phages would have no genomes and therefore could not form a plaque. We did not see any significant change after LPS incubation for either Moo19 or B2 (Fig. 8); therefore, we conclude from our collective results that LPS is necessary but not sufficient for infection by these phages. Considering that the common *Shigella* phage secondary receptors OmpA and OmpC were not critical for infection, we cannot determine which membrane protein(s) fulfill this secondary receptor role for Moo19 and/or B2. Unfortunately, no single gene deletion library or transposon library is in existence currently for CFS100. Therefore, we cannot assay all possible *omp* knockouts to conclusively determine which protein serves this function.

In summary, we have isolated and fully characterized two recently isolated *Schitoviridae* phages in terms of the capsid, tails, and receptor binding motifs. Our work reveals insight into *T* = 9 podophages, a highly understudied group within the short, noncontractile tailed phages.

## MATERIALS AND METHODS

### Phage isolation, purification, and amplification

Both phages Moo19 and B2 were isolated using previously developed methods (*24*, *25*), from Lincoln, Nebraska as part of a high school outreach activity, and identified, purified, and amplified using the following protocol. Water samples were filtered using a 0.45-μM filter, and 250 μl of the filtrate was plated on LB plates with a 0.7% soft agar overlay containing bacterial strain CFS100 *gtr*- (see tables S2 and S3 for complete list of strains). Plates were incubated overnight at 37°C and screened for plaque formation the next day. Plaques were passaged at least three times to confirm the presence of one isolated species. Phage preps from isolated plaques were then grown in a 30-ml culture of LB with 1 ml of CFS100 *gtr*- for 5 hours at 37°C while shaking at 250 rpm. The lysate was centrifuged at 4°C for 10 min at 8000*g* to remove the debris, and the supernatant was spun at 4°C for 90 min at 26,000*g*. The phage pellet was then resuspended by overnight nutation at 4°C with 2 ml of a phage dilution buffer [10 mM Tris (pH 7.6) and 10 mM MgCl_2_].

Moo19 and B2 phage stocks were further purified by cesium gradient sedimentation as previously described (*57*). Briefly, from the bottom: 1 ml of CsCl (1.6 g/cm^3^), 1 ml of CsCl (1.4 g/cm^3^), 1 ml of 25% sucrose, and 1.5 ml of a high-titer phage, all in phage dilution buffer. The gradients were spun in a Sorvall ultracentrifuge at 106,600*g* for 3 hours at 18°C. Phage bands were syringe extracted, and the samples were dialyzed against the phage dilution buffer at 4°C overnight.

### Transmission electron microscopy and 3D image reconstruction

For morphology determination, we imaged negatively stained samples. Approximately 5-μl aliquots of purified Moo19 or B2 virions were applied to glow discharged continuous carbon grids (Ted Pella, copper on Formvar, 200 mesh). Samples were briefly washed with water and stained with uranyl acetate.

Cryo-EM data were collected either at Purdue’s Cryo-EM facility using a Titan Krios equipped with a K3 direct electron detector and operating at 300 keV with a post-column Gatan Imaging Filter (GIF) (20-eV slit width) (for Moo19) or at the University of Wisconsin using a Titan Krios equipped with a K3 direct electron detector and operating at 300 keV with a post-column GIF (20-eV slit width) (for B2). For Moo19, micrographs were collected at a ×53,000 nominal magnification (0.816 Å/pixel) by recording 40 frames over 4.4 s for a total dose of 33 e^−^/Å^2^. For B2, micrographs were collected at a ×105,000 nominal magnification (0.834 Å/pixel) by recording 40 frames over 3.4 s for a total dose of 40 e^−^/Å^2^. Icosahedral and asymmetric image reconstructions were carried out as previously described (*29*). For the isolated tail proteins, gp82 and the complex of gp48/49 cryo-EM data were collected at Michigan State’s Research Technology Support Facility (RTSF) Cryo-EM facility using a Talos Arctica equipped with a Falcon 4i direct electron detector, operating at 200 keV with a post-column Selectris energy filter (10-eV slit width). Micrographs were collected at ×130,000 nominal magnification (0.886 Å/pixel) in Electron Event Representation (EER) format over 6.0 s for a total dose of 43.14 e^−^/Å^2^.

Data processing was carried out using Relion 4.0.1 for Moo19 and B2 virion. Briefly, the dose-fractionated movies were subjected to motion correction and binned 2X using Relion’s own implementation of MotionCor2. Contrast Transfer Function (CTF) estimation of the resulting images was estimated using CTFFIND-4.1, and particles were picked using the Autopick option. For the icosahedral reconstruction of the Moo19 virion capsid, 99,582 particles were used for 3D refinement, with an ab initio model serving as the initial model. For the asymmetric reconstruction of the virion, a total of 95,783 particles were used for 3D refinement, with the N4 virion map (EMD:1475, symmetrized using Relion 3.0.8) serving as an initial model. Subparticle extraction was carried by shifting the center of the particles from asymmetric refinement for the localized reconstruction of the Moo19 tail. A total of 95,783 extracted subparticles were used for 3D refinement with C6 symmetry. For the icosahedral reconstruction of the B2 virion capsid, 184,311 particles were used for 3D refinement, with an ab initio model serving as the initial model. For the asymmetric reconstruction of the B2 virion, a total of 135,384 particles were used for 3D refinement, with the Moo19 virion map serving as an initial model. Subparticle extraction was carried out by shifting the center of the particles from asymmetric refinement for the localized reconstruction of the B2 tail. A total of 133,395 extracted subparticles were used for 3D refinement with C6 symmetry. For the Gp48-Gp49 complex and Gp82, data processing was carried out using CryoSPARC 4.4.1. The micrographs were first motion corrected using patch motion correction, followed by CTF estimation using patch CTF estimation, and particles were picked using a blob picker. For the Gp48-Gp49 complex, a total of 2,078,531 particles were used for 3D refinement with C3 symmetry. For Gp82, a total of 563,035 particles were used for 3D refinement with C3 symmetry. The overall resolution was estimated based on the gold-standard Fourier shell correlation (FSC_0.143_). The final maps were deposited into the Electron Microscopy Data Bank (EMDB) (see tables S4 and S5 for accession numbers). Initial models were generated using ModelAngelo using a combination of both sequence and nonsequence modes. Refinement was carried out using Phenix, and model adjustments were carried out in COOT. Model parameters were monitored using MolProbity in Phenix, and the values are listed in tables S3 and S4 along with the respective PDB IDs.

### Host range and efficiency of plating

Initial host range results were performed by combining bacterial cells in a double agar overlay method. Once the agar containing each bacterial host solidified, 5 μl of a phage stock was applied to the top of the agar and left to dry before incubating overnight. Hosts with a positive result showed a cleared spot the next day, whereas hosts that produced a negative result had no inhibited cell growth. Following positive growth on a host, quantitative plaque assays were performed at 37°C. The efficiency of plating (EOP) was determined by calculating the titer on the experimental host and dividing by the titer on the permissive host strain, CFS100.

### Genome extraction, sequencing, and annotation

Phage genomes were extracted as previously described (*58*). The purified genomes were sequenced and assembled by the Center for Computational and Integrative Biology (CCIB) DNA Core Facility at Massachusetts General Hospital (Cambridge, MA). GeneMarkS (*59*) was used to identify open reading frames; both sequences were manually annotated using BLAST and InterPro scan (*60*) results. Any transfer RNAs were determined by tRNAscan-SE (*61*).

### Phylogenetic analysis

Phylogenetic analysis of phages was completed according to Doore *et al.* with the following changes (*25*). Sequences for alignment were obtained through GenBank selecting phages with sequence similarity according to an National Center for Biotechnology Information (NCBI) BLAST search (*62*). Whole phage genomes were aligned using MAFFT (*63*) with default settings. Next, trees were generated using MrBayes version 3.2.7a (*64*) under a mixed model for haploid genomes with gamma variation.

### Mass spectrometry

Both phages were precipitated with 10% trichloroacetic acid, and pellets were resuspended in 5 μl of a 1x SDS loading buffer and boiled at 95°C for 5 min. Samples were run on a 15% SDS gel at 200 V until the sample just passed the stacking gel into the resolving gel. The gel was then stained with Coomassie blue, and the single band containing the entire protein content from the mature virions was excised. Phages were submitted to the RTSF mass spectroscopy facility at Michigan State University, where the bands underwent a proteolytic digestion and liquid chromatography–tandem mass spectrometry analysis using a Thermo Fisher Scientific Q Exactive mass spectrometer (*65*, *66*) to aide in identifying the structural proteins and the presence of viral polymerase.

### Expression and purification of the tail proteins

Two separate vectors were generated to produce either the single gene product of gp82 from Moo19 or coexpression of two gene products from B2 (gp48 and gp49) using similar approaches as described previously (*29*). The gene encoding gp82 was amplified from Moo19 genomic DNA using primers (5′ GTGCCGCGCGGCAGCCATATGGGTATGAACTCTCACATTCCATTTGATGC 3′ and 5′ GGTGGTGGTGGTGGTGCTCGAGTTAGAAGCAACCTACAGCAGACTCAGAGTTACC 3′) and cloned into the pET28a vector (Novagen) digested with Nde I and Xho I using the Gibson assembly, which added a 6X His tag to gp82. The genes encoding gp48 and gp49 were amplified from B2 genomic DNA first using primers for gp49 (5′ CCAGGATCCGAATTCGAGCTCGATTCGAACTACAAATACGTGCTGTGGTAATCAAGC 3′ and 5′ GACTTAAGCATTATGCGGCCGCAAGCTTTTATACAACAGCACCAGTTGAGTCTACCC 3′) and cloned into the vector using Hind III and Sac I, followed by a second cloning step using gp48 (5′ GTATAAGAAGGAGATATACATATGGCCAATCAGTTATTTAGTCAAGGTGG 3′ and 5′ GCAGCGGTTTCTTTACCAGACTCGAGGTTAGATGGATGCTTCAGAGTTACC 3′) and cloned into the same vector digested with Nde I and Xho I and Xho I using the Gibson assembly. In this vector, gp49 has the 6X His tag and gp48 is untagged. Each plasmid was transformed into BL21DE3 cells for expression. Purification was performed as described previously (*29*), with no modifications.

### Colorimetric assay for esterase activity

Esterase activity experiments were performed on a Molecular Devices FilterMax F5 plate reader using 96-well plates. Each well contained a 200-μl final volume, using Buffer A [10 mM Tris (pH 7.6), 100 mM NaCl, and 10 mM MgCl_2_] along with increasing amounts of tailspike protein (ranging 0 to 10 μg) and a fixed amount of 4-nitrophenyl acetate (100 μM). Absorbance at 405 nm at room temperature was measured immediately after mixing and up to 5 min after mixing with vigorous shaking before each read. Each plate had three technical replicates per condition, and the entire experiment was repeated twice.

### LPS extraction and phage interactions

Bacterial LPS was extracted from either CFS100 or CFS100 *gtr*- using an LPS extraction kit (Bulldog Bio) as previously described (*15*, *52*). The phage and LPS were incubated for 60 min at 37°C before titering. The titer of each experimental condition was divided by the titer of the phage without LPS.

### Statistical analysis and reproducibility

All biological phage plating experiments were repeated with at least three biological replicates. Error bars reflect the SD. For the phylogenetic analysis, two replicates of tree construction were run until the SD of split frequencies was below 0.01. Trees were visualized using FigTree version 1.4.4 (*67*). For the cryo-EM data, we used a combination of programs including RELION, CryoSPARC, and Phenix (*68*–*70*). Esterase activity assays were conducted in triplicate.
