# Supplementary material for: Moo19 and B2: Structures of Schitoviridae podophages with T = 9 geometry and tailspikes with esterase activity
Source: Sci Adv. 2024 Dec 18;10(51):eadt0022. doi: 10.1126/sciadv.adt0022 (PMC11654681; doi:10.1126/sciadv.adt0022)
Supplement: Supplementary file 1 — Tables S1 to S4 [file sciadv.adt0022_sm.pdf]

Science Advances
AAAS

Supplementary Materials for

Moo19 and B2: Structures of *Schitoviridae* podophages with $T = 9$ geometry and tailspikes with esterase activity

Sundharraman Subramanian *et al.*

Corresponding author: Kristin N. Parent, kparent@msu.edu

Sci. Adv. **10**, eadt0022 (2024)
DOI: 10.1126/sciadv.adt0022

This PDF file includes:

Tables S1 to S4

Supplementary Materials

Supplementary Table 1: Bacterial Strains for Host Range Analysis

Species	Strain	Reference
Escherichia coli	REL606	Jeong et al., 2009 (71)
	BW25113	Grenier et al., 2014 (72)
	C122	Fane and Hayachi, 1991 (73)
Salmonella	MZ1597	Porwollik et al., 2014 (74)
	DB7136	Winston and Miller, 1979 (75)
Bacillus subtilis	1A1299	Borriss et al., 2017 (76); Hardwood and Wipat, 1996 (77); Kunst et al., 1997 (78)
*Shigella sonnei**	N/A	Doore et al., 2018 (24)
*Shigella boydii**	N/A	Doore et al., 2018 (24)
*Shigella dysenteriae**	N/A	Doore et al., 2018 (24)
Shigella flexneri	CFS100	Marman et al., 2014 (79)
	PE577	Macpherson et al., 1991 (80)

The following bacterial strains were used for isolation of phages and description of phage host range: *Shigella flexneri*, *Escherichia coli, Salmonella,* and *Bacillus subtilis*. Phages were initially grown in media containing 10mM calcium chloride and 10 mM magnesium chloride. An asterisk (*) indicates the pathogenic strains of *Shigella flexneri, Shigella sonnei, Shigella boydii,* and *Shigella dysenteriae*.

Supplementary Table 2 Bacterial Strains for Receptor Binding Analysis

Strain	Reference
ompA-	This study
ompC-	This study
ompA-/ompC-	This study
gtr-	Teh, M.Y. et al., 2020 (53)
gtr-/oacD-	This study
gtr-/oacB-	This study
gtr-/oacD-/oacB-	This study
oacB-	This study
wecD-	This study
wecC-	This study
waaC	This study
waaL-	This study
waaG-	This study

Bacterial knockouts made targeting the LPS and the o-antigen region of the LPS were made using lambda red recombineering techniques described in Datsenko and Wanner, 2000. Both LPS knockouts and knockouts in the o-antigen region of the LPS are further described in *Shigella flexneri* in Teh et al., 2020 (53) and Doore et al., 2021 (49), respectively. Outer membrane protein knockouts were made as described in Parent et al., 2014 (15), and Tinney et al. 2022 (26).

Supplementary Table 3: Cryo-EM data collection, refinement and validation statistics for Moo19

Map	Moo19 Capsid	Moo19 Tail	Moo19 Virion	Moo19 Gp82
EMD Identifier	EMD- 46622	EMD- 46623	EMD- 46625	EMD- 46624
PDB Identifier	9D7Z	9D80	-----	9D81
**Data collection and processing**				
Magnification	53,000	53,000	53,000	130,000
Voltage (kV)	300	300	300	200
Electron exposure (e–/Å^2)	33	33	33	43
Defocus range (μm)	-0.8 to - 3.0	-0.8 to - 3.0	-0.8 to - 3.0	-0.7 to -2.1
Pixel size (Å)	0.816	0.816	0.816	0.886
Symmetry imposed	I1	C12	C1	C3
Number of micrographs	5,241	5,241	5,241	7,726
Number of particles	95,783	95,783	95,783	563,035
Map resolution (Å)	3.6	3.7	4.3	2.4
$FSC_{0.143}$				
**Refinement**				
R.m.s. deviations				
Bond lengths (Å)	0.006	0.004	N/A	0.006
Bond angles (°)	1.042	0.683		0.888
Validation				
MolProbity score	2.28	1.72	N/A	2.08
Clashscore	9.95	5.05		14.08
Poor rotamers (%)	2.33	1.27		1.41
Ramachandran plot				
Favored (%)	92.19	94.46	N/A	95.51
Allowed (%)	7.64	5.16		3.85
Disallowed (%)	0.17	0.38		0.64
CC (volume)	0.80	0.71	N/A	0.92

N/A = Not Applicable as there is no associated PDB file

Supplementary Table 4: Cryo-EM data collection, refinement and validation statistics for B2

Map	B2 Capsid	B2 Tail	B2 Virion	B2 Gp82
EMD Identifier	EMD- 46626	EMD- 46627	EMD- 46629	EMD- 46628
PDB Identifier	9D82	9D83	---	9D84
**Data collection and processing**				
Magnification	105,000	105,000	105,000	130,000
Voltage (kV)	300	300	300	200
Electron exposure (e–/Å^2)	40	40	40	43
Defocus range (µm)	-0.5 to -2.0	-0.5 to -2.0	-0.5 to -2.0	-0.7 to -2.1
Pixel size (Å)	0.834 (unbinned)	0.834 (unbinned)	0.834 (unbinned)	0.886
Symmetry imposed	I1	C12	C1	C3
Number of micrographs	12,799	12,799	12,799	6,456
Number of particles	135,384	135,384	135,384	2,078,531
Map resolution (Å)	3.4	3.5	4.1	2.3
FSC threshold 0.143				
**Refinement**				
R.m.s. deviations				
Bond lengths (Å)	0.004	0.004	N/A	0.005
Bond angles (°)	0.804	0.694		0.733
Validation				
MolProbity score	2.34	1.90	N/A	1.90
Clashscore	16.59	7.18		12.17
Poor rotamers (%)	1.33	1.46		1.35
Ramachandran plot				
Favored (%)	90.71	94.40	N/A	96.78
Allowed (%)	8.59	5.14		3.10
Disallowed (%)	0.70	0.47		0.12
CC (volume)	0.85	0.73	N/A	0.91

N/A = Not Applicable as there is no associated PDB file
